# 167 Holistic wellbeing in older persons with a chronic lung disease: The communication about sexuality (COSY) randomized controlled trial (RCT)

**DOI:** 10.1093/eurpub/ckae114.172

**Published:** 2024-09-26

**Authors:** Anja Frei, Kaba Dalla Lana, Thomas Radtke, Julia Braun, Milo Puhan, Claudia Steurer-Stey

**Affiliations:** Epidemiology, Biostatistics and Prevention Institute, University of Zurich, Zurich, Switzerland; Epidemiology, Biostatistics and Prevention Institute, University of Zurich, Zurich, Switzerland; Epidemiology, Biostatistics and Prevention Institute, University of Zurich, Zurich, Switzerland; Epidemiology, Biostatistics and Prevention Institute, University of Zurich, Zurich, Switzerland; Epidemiology, Biostatistics and Prevention Institute, University of Zurich, Zurich, Switzerland; Epidemiology, Biostatistics and Prevention Institute, University of Zurich, Zurich, Switzerland

## Abstract

**Purpose:**

Experiencing a fulfilling sexuality is important for human wellbeing. However, problems with sexuality are common in older and chronically ill people like persons with COPD, and the topic is rarely addressed by health care professionals (HCP). The aim of this RCT was to evaluate acceptability and effectiveness of the COmmunication about SexualitY (COSY) intervention on health outcomes and physical activity in people with COPD (NCT05696730).

**Methods:**

Persons with COPD recruited from general population and primary care setting were randomized into intervention (IG; 1 face-to-face and 2 phone counselling sessions by trained HCP, 6 communication instruments) or control group (CG; usual care). Primary outcome was change in quality of life (CASP-12) from baseline to 3 months follow-up, secondary outcomes change in physical activity (accelerometer, ActiGraph), functional exercise capacity, and COPD specific health outcomes. Acceptability of the IG participants was assessed by interviews at the end of the study.

**Results:**

36 persons were randomized (17 IG, 19 CG), 44.4% (n = 16) females, mean (SD) age 71.1 (6.9) years. They conducted on average 6074 (4043) step per day and 39 (35) minutes in moderate/vigorous physical activity. 27 participants (13 IG) already completed 3-months follow-up (assessments still running, effectiveness results expected by end of April 2024). So far, 54% of the interviewed IG participants stated that they were very happy and 46% that they were happy with the intervention (5-point Likert scale, 1=very happy to 5=not happy at all). They reported that the COSY instruments helped them to discover and discuss needs, goals, and barriers for sexual wellbeing, to support motivation to conduct individually defined actions for communication about their needs and for healthy behaviours.

**Conclusions:**

Preliminary results showed that communication about sexual wellbeing was well accepted by these older, chronically ill persons. Successful implementation requires not only a competent, but also an open and respectful HCP. Effectiveness results regarding health outcomes and physical activity are not available yet but will be presented at the conference.

**Funding:**

The project was funded by the foundation LUNGE ZURICH (main funding source) and AstraZeneca and OM Pharma (additional funding sources).

